# Knee Joint Loading During Supported Standing in Children and Adolescents with Severe Cerebral Palsy: Effects of Verticalization and Joint Position

**DOI:** 10.3390/children13040497

**Published:** 2026-04-01

**Authors:** René Althaus, Eva M. Steindl

**Affiliations:** 1 Independent Researcher, Auf der Seppe 19, 32689 Kalletal, Germany; 2 Independent Researcher, Schulstraße 5, 3300 Amstetten, Austria; eva.m.steindl@gmail.com

**Keywords:** cerebral palsy, supported standing, knee joint loading, verticalization angle, GMFCS, pediatric orthopaedics, hip and knee flexion, standing therapy

## Abstract

**Highlights:**

**What are the main findings?**

- Knee joint loading increases progressively as verticalization increases across all examined hip/knee positions.
- The relationship between hip/knee flexion and knee joint loading is non-linear, with the lowest loading observed at approximately 15° flexion.

**What are the implications of the main findings?**

- Knee joint loading during supported standing is influenced by both verticalization angle and joint configuration, indicating that positioning strategies can modulate mechanical knee joint loading.
- Moderate hip/knee flexion may correspond to relatively low mechanical forces at the knee and, in certain configurations, represent a mechanically favorable joint position during supported standing.

**Abstract:**

Background: Supported standing is widely used in children and adolescents with severe cerebral palsy (CP) as part of rehabilitation programs aimed at maintaining musculoskeletal health and enabling participation. Despite its frequent clinical use, quantitative biomechanical evidence describing knee joint loading under different positioning conditions remains limited, particularly in individuals classified as GMFCS IV–V. The primary objective of this study was to quantify knee joint loading during supported standing across predefined combinations of verticalization angle and hip/knee flexion. The secondary objective was to investigate interaction effects between these variables and to assess whether increasing hip/knee flexion is associated with a linear reduction in knee joint loading. Methods: Twenty-six children and adolescents with CP (GMFCS IV–V; age 6–17 years) participated in the study. Measurements were performed using a standardized back-supported standing device. Knee joint loading was measured using integrated pressure sensors across six verticalization angles (0°, 30°, 45°, 60°, 75°, 90°) combined with four hip/knee flexion angles (0°, 15°, 30°, 45°). Forces were normalized to body weight (%BW). Statistical analysis was performed using repeated-measures analysis of variance. Results: Knee joint loading increased consistently with greater verticalization across all tested hip/knee flexion conditions (*p* < 0.001). A non-linear pattern was observed across flexion angles. Interaction effects between verticalization and hip/knee flexion were observed. Knee joint loading did not decrease linearly with increasing flexion; instead, the lowest loading was observed at approximately 15° hip/knee flexion, whereas both full extension and 45° flexion resulted in higher loads. Conclusions: Verticalization angle represents a key factor influencing knee joint loading during supported standing in children and adolescents with severe CP. Knee joint loading increases with greater verticalization, while hip/knee position shows a non-linear influence. The absence of a linear reduction in loading with increasing flexion highlights the presence of interaction effects between positioning variables and supports individualized positioning strategies in supported standing programs.

## 1. Introduction

The clinical presentation of cerebral palsy (CP) varies widely in severity and functional impairment and is commonly classified using the Gross Motor Function Classification System (GMFCS) [1]. This five-level system differentiates motor function, with levels IV–V representing children and adolescents with severe limitations in self-mobility: while individuals at level IV may achieve limited mobility with assistive devices, those at level V are fully dependent on external support for positioning and mobility.

In this population, supported standing represents a key therapeutic intervention, as independent upright posture and physiological weight-bearing of the lower extremities cannot be achieved. However, its clinical implementation is challenging due to pronounced motor impairment, altered muscle tone regulation, and frequently present joint contractures, requiring carefully individualized positioning strategies.

Recent research further highlights the multifactorial etiology of CP, including genetic contributions and neuroinflammatory mechanisms, expanding the traditional understanding of its pathophysiology [2,3,4,5,6,7]. Previous studies have demonstrated that supported standing is associated with positive effects on bone mineral density, gastrointestinal function, joint mobility, respiratory performance, and participation in daily activities [8].

Nevertheless, quantitative evidence describing the biomechanical loading during supported standing remains limited. Adequate mechanical stimulation of the lower extremities is considered essential for maintaining bone integrity, joint alignment, and musculoskeletal function. Children and adolescents with severe CP frequently present with structural and functional characteristics such as hip and knee flexion contractures, altered muscle tone, and reduced extensor strength, all of which may substantially influence load transmission during supported standing [9,10].

While previous studies have primarily focused on plantar loading or general weight-bearing outcomes [11,12,13], data on joint-specific loading—particularly at the knee—remain scarce. In particular, the combined effects of verticalization angle and hip/knee flexion on knee joint loading have not yet been systematically investigated, despite their direct clinical relevance for optimizing positioning strategies in children with limited range of motion or joint contractures.

To our knowledge, this study is the first to quantitatively assess knee joint loading across systematically combined verticalization angles and hip/knee flexion positions in children and adolescents with severe cerebral palsy (GMFCS IV–V). Against this background, the present study aims to quantify knee joint loading in children and adolescents with severe CP (GMFCS IV–V) across a standardized matrix of verticalization angles and hip/knee flexion positions using integrated pressure sensor technology. By analyzing interaction effects between positioning parameters and testing the assumption of a linear relationship between increasing hip/knee flexion and load reduction, this study extends current biomechanical understanding beyond descriptive loading patterns toward clinically applicable positioning principles.

Building on our previous investigation of plantar loading, in which foot weight-bearing was quantified as a percentage of body weight under identical experimental conditions, the present study focuses on knee joint loading within the same cohort and experimental framework [14]. This approach enables a direct comparison of load distribution across different segments of the lower extremities, taking into account their distinct biomechanical functions.

In this context, the study was designed as an exploratory cross-sectional investigation using a within-subject design.

The primary objective was to quantify knee joint loading in children and adolescents with severe CP (GMFCS IV–V) across systematically varied combinations of verticalization angles and hip/knee flexion positions during supported standing.

The secondary objective was to investigate interaction effects between verticalization angle and hip/knee flexion and to assess whether increasing flexion is associated with a linear reduction in knee joint loading.

## 2. Materials and Methods

### 2.1. Study Design and Reporting

The present investigation was conducted as an exploratory cross-sectional study in accordance with the Strengthening the Reporting of Observational Studies in Epidemiology (STROBE) recommendations. Reporting followed established STROBE guidelines for observational research, where applicable.

To evaluate the effects of positioning variables, a within-subject repeated-measures framework was implemented, allowing each participant to serve as their own control across all tested conditions.

### 2.2. Participants and Eligibility Criteria

A total of 26 children and adolescents with a confirmed diagnosis of CP (GMFCS levels IV–V), aged 6 to 17 years, were included in the study. The cohort comprised 15 children (6–12 years) and 11 adolescents (13–17 years). Detailed demographic and clinical characteristics are presented in Table 1.

The investigated cohort corresponds to that previously analyzed in a prior study on plantar loading during supported standing [14]. Although data collection was performed using the same standardized protocol, the datasets were analyzed separately to address distinct biomechanical outcome measures at the foot and knee levels. The decision to report plantar and knee joint loading in separate manuscripts was made to ensure methodological clarity and to allow a focused analysis of distinct biomechanical constructs with different clinical implications.

While plantar loading primarily reflects vertical force transmission to the foot, the present study considers knee joint loading as a distinct biomechanical construct. In addition to vertical load transfer, it is influenced by joint alignment, lever arm mechanics, and the interaction between verticalization angle and hip/knee flexion. These factors are particularly relevant in the context of contractures and individualized positioning strategies in severely affected patients.

Inclusion and exclusion criteria are summarized in Table 2.

### 2.3. Recruitment

Recruitment was conducted between January and February 2025 at specialized educational and therapy centers in Germany. Identification of eligible participants and preselection were carried out by the treating therapists based on predefined inclusion and exclusion criteria.

Legal guardians received written information about the study and were invited to provide informed consent for participation. As recruitment was conducted exclusively through specialized centers and not population-based, selection bias cannot be entirely excluded. All measurements were performed on-site using a standardized standing system.

### 2.4. Equipment and Measurement System

**Figure 1:**
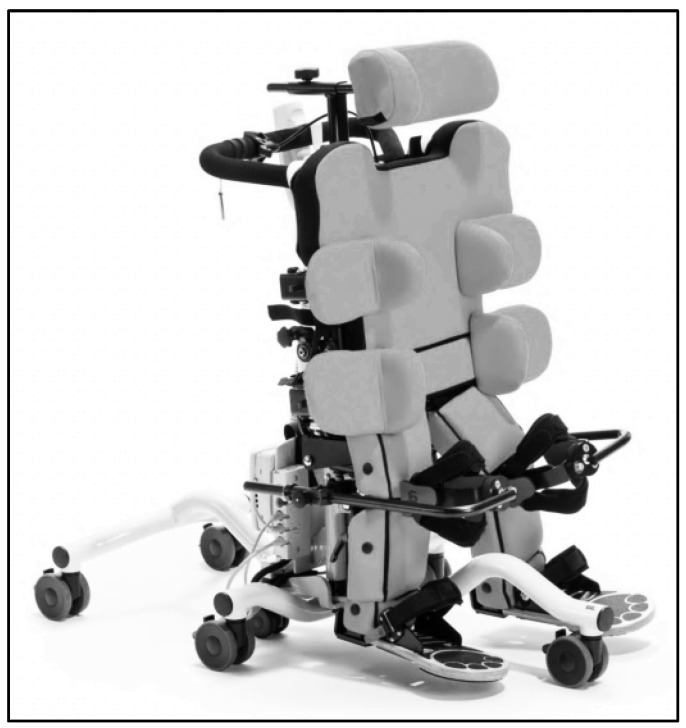
Till-supported standing system. Image provided by Schuchmann GmbH & Co. KG, Germany.

Measurements were conducted using a back-supported standing system (Till, Schuchmann GmbH & Co. KG, Bissendorf, Germany), which allows controlled adjustment of the verticalization angle as well as hip and knee flexion, while providing continuous stabilization of the trunk and lower extremities (Figure 1).

Body weight was determined prior to data collection using a calibrated scale integrated into a patient lift (Arnold 150, Rebotec, Germany) and served as the reference value for normalization of the measured forces. Calibration of all measurement systems was performed before each data collection session using standardized reference weights.

**Figure 2:**
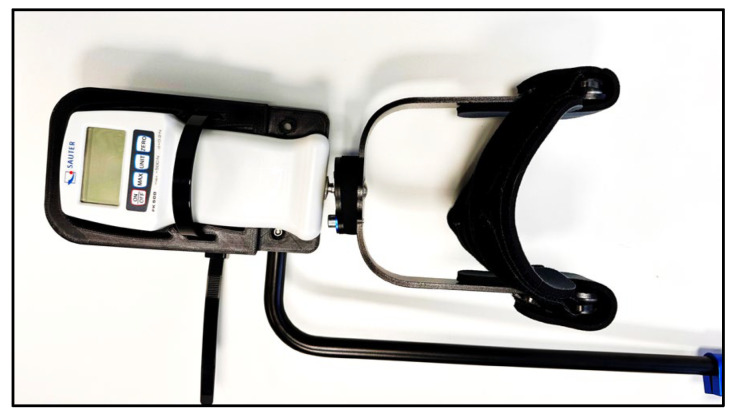
Pressure Sensor Sauter FK500; image provided by Schuchmann GmbH & Co. KG, Germany.

Knee joint loading was assessed using force sensors (Sauter FK500, Kern & Sohn GmbH, Balingen, Germany) integrated into the anterior knee supports. These sensors recorded externally applied forces transmitted to the ventral aspect of the knee via the standing system, without direct loading of the patella (Figure 2).

In this study, the term “knee joint loading” refers to these externally measured force components and does not represent internal tibiofemoral joint contact forces.

The lower extremities were positioned symmetrically, with particular attention to proper alignment of the hip, knee, and ankle joints. The feet were placed flat on the footplates and aligned approximately perpendicular to the lower legs. If required, individually fitted orthoses or orthopedic footwear were used to ensure a standardized biomechanical baseline position.

The verticalization angle was determined using a digital inclinometer application (Wasserwaage + Winkelmesser App, version 1.3.8; WHATSTICKER APPS SRL, Milano, Italy), while hip and knee angles were measured with a goniometer along the respective joint axes.

**Figure 3:**
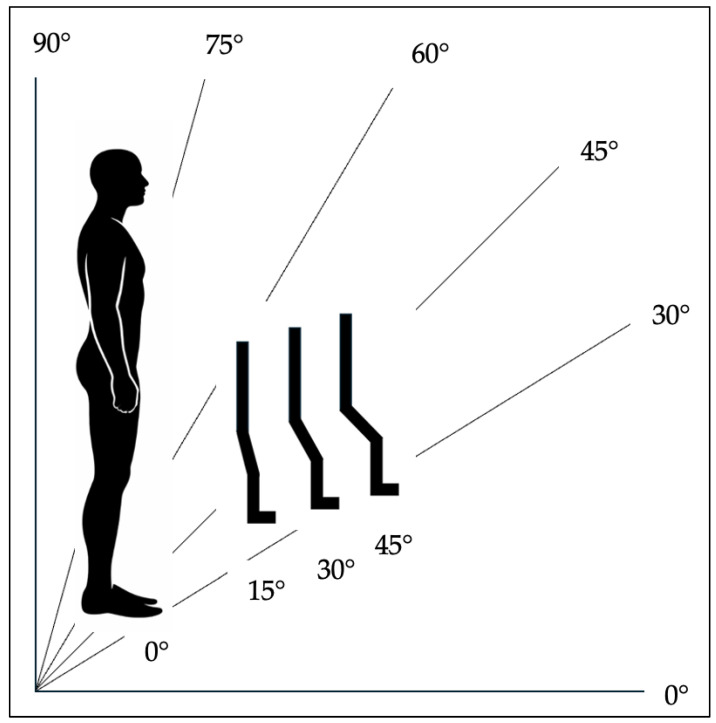
Schematic illustration of the tested verticalization angles and corresponding hip/knee flexion positions used during the examination. Source: authors’ own illustration.

A predefined set of positioning conditions was systematically tested (Figure 3), including verticalization angles of 0°, 30°, 45°, 60°, 75°, and 90°, combined with hip/knee flexion angles of 0°, 15°, 30°, and 45°.

### 2.5. Experimental Procedure and Positioning

**Figure 4:**
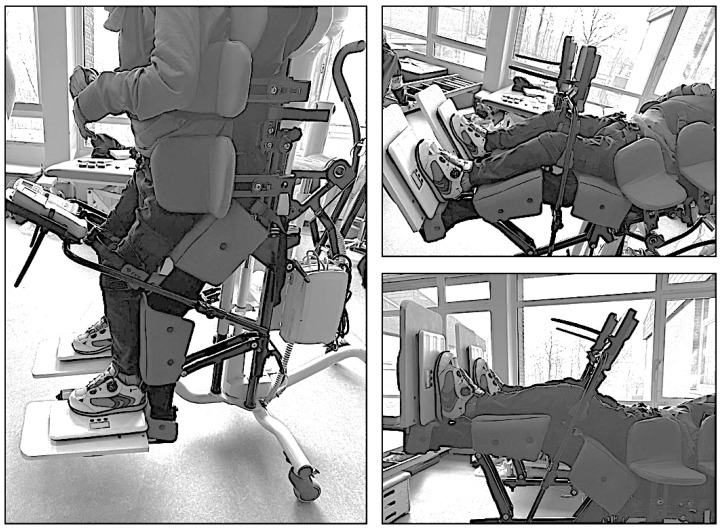
Positioning examples for HK 30° and V 90°, 30°, 0°. Source: Authors’ own photograph [14].

Positioning was performed according to a standardized protocol. Starting from a horizontal baseline position, during which transfer and instrument setup were completed, the standing system was gradually adjusted to the predefined target verticalization angles (Figure 4).

Six verticalization levels (0°, 30°, 45°, 60°, 75°, and 90°) were investigated, each combined with four hip/knee flexion positions (0°, 15°, 30°, and 45°). The sequence of test conditions was identical for all participants.

Throughout the entire procedure, trained therapists continuously monitored joint alignment, muscle tone, involuntary movements, and overall tolerance to ensure participant safety and well-being.

Prior to each measurement, an adaptation period of approximately 30 s was allowed to ensure a stable baseline condition. Data acquisition was initiated only after confirmation of a reproducible position. In cases of instability or increased muscle activity, measurements were repeated. Data sets with artifacts or insufficient stability were excluded.

To minimize fatigue effects, sufficient rest periods were provided between test conditions. No adverse events or clinically relevant discomfort occurred during the study.

Knee joint loading was expressed as a percentage of body weight (%BW). For this purpose, force values obtained from the integrated knee pressure sensors were normalized to each participant’s body weight, which had been determined beforehand using the calibrated scale integrated into the patient lift (Table 3).

### 2.6. Outcome Measures and Data Processing

Measurement data were exported, and mean values were calculated for each predefined experimental condition. Knee joint loading (%BW) was determined by normalizing the pressure-derived force values obtained from the integrated knee sensors to each participant’s individually measured body weight.

Recordings affected by motion artifacts, insufficient postural stability, excessive involuntary muscle activity, or incomplete data capture were excluded from further analysis.

Data collection was conducted between January and February 2025.

### 2.7. Statistical Analysis

Statistical analyses were performed using JASP (Version 0.18.0) and DATAtab (DATAtab e.U., Graz, Austria, available at http://datatab.net; accessed on 3 March 2025).

The effect of verticalization angle on knee joint loading was analyzed separately for each hip/knee flexion condition using one-way repeated-measures analysis of variance (ANOVA). Differences between flexion conditions were analyzed descriptively.

In cases where the assumption of sphericity was violated, Greenhouse–Geisser corrections were applied. The level of significance was set at *p* < 0.05. Effect sizes were reported as partial eta squared (ηp^2^).

To assess statistical sensitivity, a post hoc power analysis was conducted using G*Power (Version 3.1). A conservative scenario was assumed, using the smallest available sample size (*n* = 21) and the lowest observed Greenhouse–Geisser correction factor (ε = 0.28). Assuming a correlation of r = 0.50 among repeated measures, a significance level of α = 0.05, and a statistical power of 0.80, the minimum detectable effect size was f = 0.37 (ηp^2^ = 0.12).

## 3. Results

Across all tested hip/knee flexion conditions, knee joint loading showed a progressive increase with greater verticalization angles. Separate one-way repeated-measures ANOVAs demonstrated a significant effect of verticalization in all flexion conditions (all *p* < 0.001), with large effect sizes (ηp^2^ = 0.64–0.83). Descriptive statistics (mean ± SD and 95% confidence intervals) are presented in Table 4.

**Figure 5:**
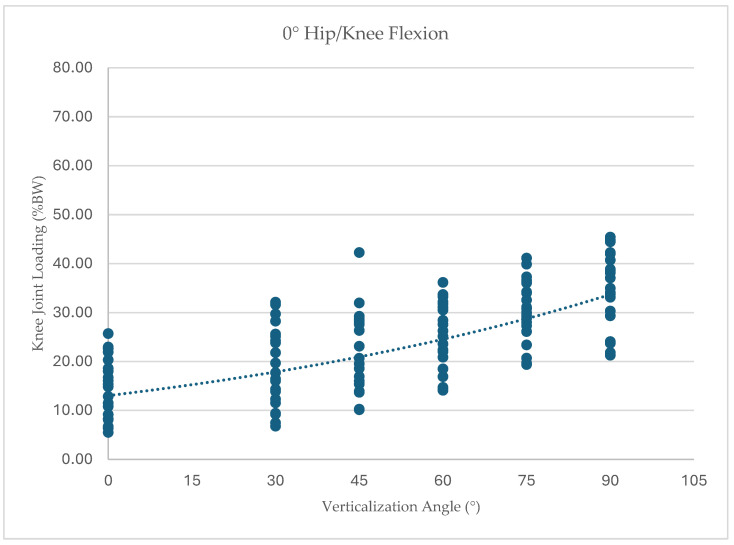
Knee joint loading (% body weight) across verticalization angles at 0° hip/knee flexion.

**Figure 6:**
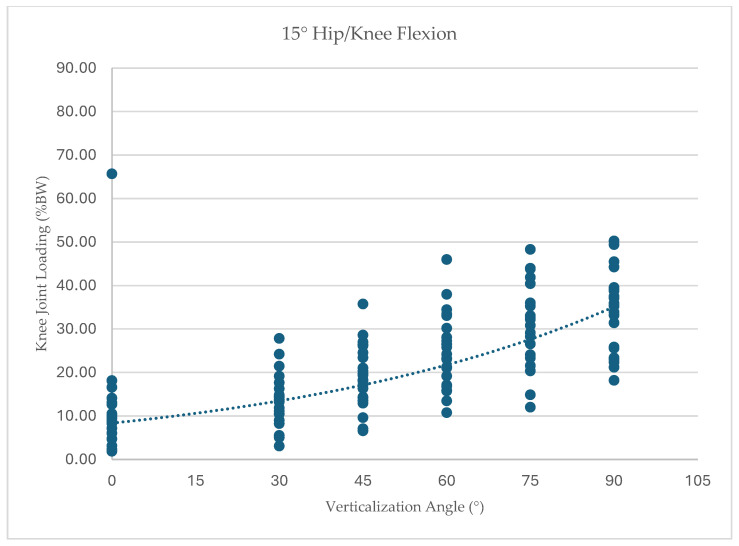
Knee joint loading (% body weight) across verticalization angles at 15° hip/knee flexion.

**Figure 7:**
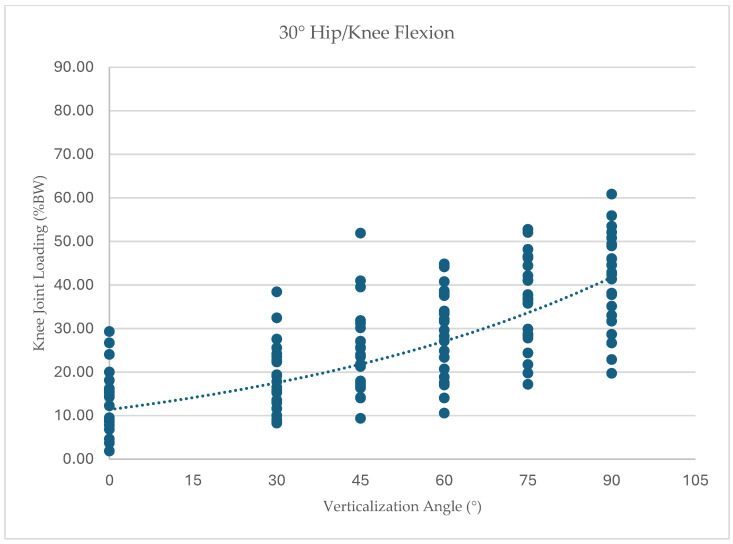
Knee joint loading (% body weight) across verticalization angles at 30° hip/knee flexion.

**Figure 8:**
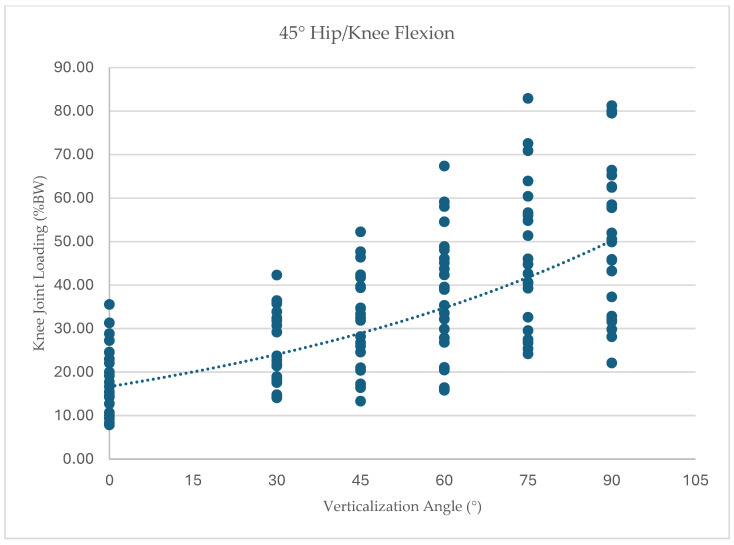
Knee joint loading (% body weight) across verticalization angles at 45° hip/knee flexion.

To further illustrate the distribution of individual responses, knee joint loading values for each participant across all tested conditions are presented in Figure 5, Figure 6, Figure 7 and Figure 8. Dots represent individual participant measurements at each verticalization angle. The dashed line represents the linear trend (best-fit regression line) illustrating the overall relationship between verticalization angle and knee joint loading.

**Figure 9:**
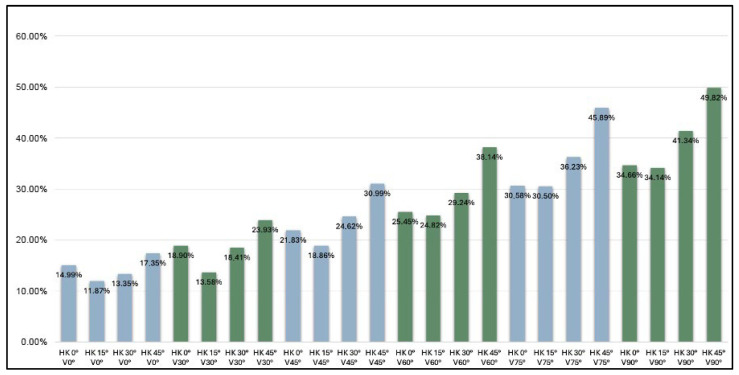
Percentage of knee joint loading (%BW) across verticalization angle and hip/knee flexion conditions.

In addition to verticalization, hip/knee flexion was associated with differences in knee joint loading. Across all verticalization levels, the lowest loading values were consistently observed at 15° flexion, whereas higher values were recorded at 0° and 30° flexion and were highest at 45° flexion, indicating a non-linear association between joint position and knee joint loading (Figure 9).

At 0° hip/knee flexion (*n* = 21), knee joint loading increased from 14.99% BW (SD 6.29) at 0° verticalization to 34.66% BW (SD 7.64) at 90° verticalization. Because the assumption of sphericity was violated, Greenhouse–Geisser corrections were applied (ε = 0.28). A significant effect of verticalization was observed, F(1.40, 28.0) = 99.54, *p* < 0.001, ηp^2^ = 0.83.

At 15° hip/knee flexion (*n* = 23), loading increased from 11.87% BW (SD 12.72) at 0° verticalization to 34.14% BW (SD 9.06) at 90° verticalization, F(1.39, 30.6) = 39.86, *p* < 0.001, ηp^2^ = 0.64.

At 30° hip/knee flexion (*n* = 23), knee joint loading increased from 13.35% BW (SD 7.09) at 0° verticalization to 41.34% BW (SD 10.74) at 90° verticalization, F(1.39, 30.6) = 85.14, *p* < 0.001, ηp^2^ = 0.79.

At 45° hip/knee flexion (*n* = 25), loading increased from 17.35% BW (SD 8.17) at 0° verticalization to 49.82% BW (SD 16.89) at 90° verticalization, F(1.40, 33.6) = 90.88, *p* < 0.001, ηp^2^ = 0.79.

Across flexion conditions, increasing hip/knee flexion did not result in a linear reduction in knee joint loading. At 90° verticalization, loading was 34.66% BW at 0° flexion, 34.14% BW at 15° flexion, 41.34% BW at 30° flexion, and 49.82% BW at 45° flexion, indicating a non-linear relationship between joint position and knee joint loading.

Overall, both verticalization angle and hip/knee flexion emerged as key determinants of knee joint loading.

Considerable inter-individual variability in knee joint loading was observed across participants within all tested conditions. While group-level trends showed consistent increases in loading with higher verticalization angles, individual responses varied substantially, particularly at higher degrees of hip/knee flexion. These findings indicate that knee joint loading cannot be fully explained by positioning parameters alone and support the need for individualized assessment and positioning strategies in supported standing.

## 4. Discussion

To our knowledge, this study represents one of the first quantitative investigations specifically addressing knee joint loading during supported standing in children and adolescents with severe CP. The findings demonstrate that both the degree of verticalization and the selected joint configuration significantly influence the magnitude of mechanical knee joint loading.

Across all tested configurations, knee joint loading increased consistently with increasing verticalization. Higher verticalization angles were therefore associated with greater mechanical stress at the knee joint, largely independent of the respective flexion angle. This observation is biomechanically plausible, as progressive verticalization increases the proportion of body weight transmitted through the lower extremities, thereby elevating the axial compressive load acting on the knee joint.

A non-linear relationship was identified between hip/knee flexion and knee joint loading, with a distinct load minimum at approximately 15° of flexion. Loading values were higher both in full extension and at greater flexion angles (30° and 45°). This pattern can be explained by changes in lever arm mechanics and the orientation of the ground reaction force. At slight flexion, the line of action of the ground reaction force passes closer to the knee joint center, reducing the external flexion moment. At the same time, moderate flexion allows stable tibiofemoral load distribution without requiring substantial internal extension moments. With increasing flexion, however, the lever arm of the ground reaction force relative to the knee joint center increases, necessitating higher internal extension moments and resulting in elevated tibiofemoral compressive forces.

A multifactorial contribution to the observed loading patterns must also be considered. Flexion-dependent variations in muscle tone and co-contraction, differences in tibiofemoral contact mechanics, and individual structural factors such as contractures or malalignment—commonly present in children with CP—may additionally influence load distribution [15].

The highest loading values were observed at 45° hip/knee flexion combined with full verticalization (90°), reaching a mean of 49.82% of body weight. In contrast, the lowest values consistently occurred at approximately 15° of flexion—even at maximum verticalization—and were lower than those observed in full extension. These findings suggest that moderate flexion positions may, under certain conditions, represent mechanically favorable joint configurations compared with full extension.

The present findings complement existing literature by providing insights into knee joint loading under controlled static positioning conditions. The present study extends existing knowledge by providing segment-specific insights into knee joint loading under controlled static positioning conditions. These findings are particularly relevant for individuals with severe motor impairment, in whom mechanical loading is largely dependent on externally guided positioning rather than active movement. By quantifying knee joint loading under standardized positioning conditions, the present study contributes to a more differentiated understanding of load distribution across the lower extremities during supported standing. These findings are consistent with observations in non-ambulatory populations, in which externally supported standing represents a primary source of mechanical loading in the absence of independent mobility.

In contrast to dynamic activities such as walking or running, which are characterized by substantially higher internal joint contact forces, the present study assessed externally transmitted forces at the anterior knee interface under static supported standing conditions. These fundamentally different biomechanical contexts limit direct comparability with gait-related loading data and should be interpreted as complementary rather than equivalent measures of joint loading.

The knee joint loads measured in this study remained within a moderate range relative to reported static loading conditions, and no indications of acute clinical overload were observed. Neither verbal nor nonverbal signs of pain or discomfort were noted in any of the tested positions [9].

It must be emphasized that the present study quantified externally transmitted vertical support forces measured at the anterior knee interface rather than internal tibiofemoral joint forces. Internal joint contact forces are known to substantially exceed externally measured load components due to muscle co-contraction and joint reaction forces. Furthermore, measurements were obtained under static supported standing conditions and therefore cannot be directly compared with dynamic joint loading reported during gait or other functional activities. Consequently, comparisons with literature-derived tibiofemoral contact forces must be interpreted with caution [16].

### 4.1. Clinical Implications

The present results have several clinical implications. Children and adolescents with hip/knee flexion contractures were able to achieve measurable knee joint loading under the tested positioning conditions. Knee joint loading was significantly influenced by both verticalization angle and hip/knee flexion, with lower loading observed at approximately 15° of flexion compared with full extension or greater flexion angles. Importantly, the identification of a load minimum at approximately 15° flexion provides a clinically actionable parameter that may support targeted positioning strategies in supported standing.

These findings indicate that individualized configuration of supported-standing systems, taking anatomical alignment and available joint range of motion into account, may help clinicians modulate mechanical knee joint loading during supported standing. Accordingly, both the achievable level of verticalization and existing movement limitations should be carefully considered when prescribing and adjusting standing devices.

Given the considerable clinical heterogeneity within this population and the frequently limited ability of patients to report discomfort, attentive clinical monitoring during supported standing remains essential.

The present study provides novel quantitative data on externally measured knee joint loading under controlled supported standing conditions in children with severe CP. By identifying verticalization angle and joint configuration as key determinants of loading, the findings offer clinically relevant guidance for optimizing positioning strategies in non-ambulatory individuals. This is particularly relevant in clinical practice, where positioning decisions are often based on empirical experience rather than objective biomechanical data.

### 4.2. Strengths and Limitations

A key strength of the present study is the systematic evaluation of 24 predefined combinations of verticalization angles and hip/knee flexion positions, which enabled a structured examination of how positioning influences knee joint loading. The use of a standardized positioning protocol, together with force measurement integrated directly into the supported-standing device, ensured consistent measurement conditions and supported reproducible data collection. In addition, the study specifically examined children and adolescents with severe CP (GMFCS levels IV–V), a population that remains relatively underrepresented in biomechanical investigations.

However, several limitations should be acknowledged. First, the analysis was limited to static standing configurations, and therefore dynamic changes occurring during transitions between different verticalization angles were not evaluated. Consequently, the present data reflect static load conditions only and do not capture dynamic joint loading patterns encountered during positional changes. Second, mediolateral force distribution and potential asymmetries were not analyzed, and no bilateral side-specific evaluation was performed. This may have limited the detection of asymmetrical loading patterns.

Third, knee joint loading was assessed using externally measured vertical force components applied ventrally at the knee joint via a pressure sensor system integrated into the knee support pad. Internal tibiofemoral contact forces were not directly measured; furthermore, neither inverse dynamic modeling nor biomechanical simulation of internal joint reaction forces was performed. In addition, no electromyographic (EMG) recordings were obtained, precluding analysis of muscle activation patterns and co-contraction effects. The pressure sensor system used for load quantification was not formally validated against a gold-standard reference method, which may potentially affect measurement accuracy.

Although no a priori power calculation was performed, a post hoc sensitivity analysis demonstrated sufficient statistical power to detect medium to large main effects of verticalization within the repeated-measures design. The observed effect sizes (ηp^2^ = 0.64–0.83) were well above the minimum detectable threshold. However, interaction effects between verticalization angle and hip/knee flexion were not formally tested within the chosen analytical approach. Consequently, potential interaction patterns should be interpreted descriptively rather than inferentially.

### 4.3. Future Research

Future studies should employ longitudinal designs to explore how repeated supported standing interventions influence knee joint loading over time and to determine whether sustained use leads to measurable changes in loading patterns or other joint-related parameters. The influence of orthotic use, pre-existing hip/knee flexion contractures, and variations in standing system configuration on knee joint loading patterns warrants systematic evaluation.

Furthermore, dynamic transitions between verticalization and varying hip/knee flexion and abduction positions should be examined to better reflect clinically relevant loading conditions encountered in routine practice.

Given the exploratory nature of the present study as well as the relatively small and clinically heterogeneous sample, confirmation of the present findings in larger, controlled cohorts appears warranted.

## 5. Conclusions

Higher verticalization angles during supported standing were associated with a significant increase in externally measured knee joint loading in children and adolescents with severe CP (GMFCS IV–V). Hip/knee flexion angles substantially influenced load magnitude, with moderate flexion (~15°) associated with lower loading compared with full extension or greater flexion in several configurations.

Within the tested static conditions, externally transmitted vertical knee forces remained measurable despite limited hip/knee extension, indicating that flexion contractures do not inherently prevent mechanical load transfer during supported standing. However, the present data reflect externally measured force components only and do not permit conclusions regarding internal tibiofemoral contact forces, clinical tolerability, or long-term joint safety.

These findings underscore the importance of individualized, axis-aligned adjustment of standing systems. Consideration of both verticalization angle and hip/knee joint position may help modulate mechanical knee joint loading during supported standing in this vulnerable population. Furthermore, the integration of appropriate measurement systems into standing devices may be beneficial to enable the assessment of individual loading conditions in clinical practice and to allow targeted repositioning in order to minimize potential pressure peaks for the respective child or adolescent.

## Figures and Tables

**Table 1:** Demographic and Clinical Characteristics of Children and Adolescents with CP (GMFCS IV–V) (*n* = 26).

Patient	Age	Sex	GMFCS	Body Weight (kg)
1	6	W	V	15.6
2	9	W	V	22
3	9	W	V	18.9
4	10	M	V	20.1
5	14	W	IV	44.5
6	16	W	V	36.2
7	11	M	IV	29.5
8	7	M	V	25.7
9	14	M	V	33
10	9	W	IV	31
11	11	W	V	23.8
12	10	W	V	26.3
13	9	W	IV	29.6
14	14	M	V	30.1
15	14	M	IV	32.9
16	15	M	V	38
17	6	W	IV	19.3
18	14	W	V	30.5
19	13	M	V	30.5
20	11	M	IV	20.9
21	9	W	V	34.8
22	17	M	V	47
23	14	M	V	40.7
24	7	M	IV	25.7
25	9	W	IV	32.2
26	16	M	V	25.75

**Table 2:** Inclusion and exclusion criteria.

Inclusion Criteria	Exclusion Criteria
Diagnosis of cerebral palsy (CP)	Other neurological disease
GMFCS Level IV or V	GMFCS Level I–III
Participation in a regular supported standing program	No regular participation in supported standing therapy
Age between 4 and 18 years	Age < 4 years or >18 years
	Inability to comply with study procedures
	Botulinum toxin injections to the lower extremities within the previous 6 months
	Orthopedic surgery of the lower extremities within the previous 6 months
	Acute medical condition at the time of assessment
	Acute pain affecting standing tolerance or knee joint loading

**Table 3:** Positions and corresponding hip/knee flexion and verticalization angle [14].

Position	Hip/Knee Flexion	Verticalization Angles
1	0°/0°	0°, 30°, 45°, 60°, 75°, 90°
2	15°/15°	0°, 30°, 45°, 60°, 75°, 90°
3	30°/30°	0°, 30°, 45°, 60°, 75°, 90°
4	45°/45°	0°, 30°, 45°, 60°, 75°, 90°

**Table 4:** Knee Joint Loading (% Body Weight) Across Verticalization Angles and Hip/Knee Flexion Conditions.

Hip/Knee Flexion	Verticalization (°)	Mean (%BW)	SD	95% CI
0° (*n* = 21)	0	14.99	6.29	12.30–17.68
	30	18.90	8.09	15.44–22.36
	45	21.83	8.50	18.19–25.47
	60	25.45	6.96	22.47–28.43
	75	30.58	6.35	27.86–33.30
	90	34.66	7.64	31.39–37.93
15° (*n* = 23)	0	11.87	12.72	6.67–17.07
	30	13.58	5.88	11.17–15.99
	45	18.86	7.20	15.92–21.80
	60	24.82	8.49	21.36–28.28
	75	30.50	9.33	26.69–34.31
	90	34.14	9.06	30.44–37.84
30° (*n* = 23)	0	13.35	7.09	10.45–16.25
	30	18.41	7.79	15.23–21.59
	45	24.62	9.84	20.61–28.63
	60	29.24	9.73	25.27–33.21
	75	36.23	10.10	32.11–40.35
	90	41.34	10.74	36.96–45.72
45° (*n* = 25)	0	17.35	8.17	14.15–20.55
	30	23.93	8.69	20.52–27.34
	45	30.99	10.69	26.80–35.18
	60	38.14	13.54	32.84–43.44
	75	45.89	15.87	39.67–52.11
	90	49.82	16.89	43.20–56.44

## Data Availability

The data supporting the findings of this study are available from the corresponding author upon reasonable request. Access to the data is restricted due to ethical considerations related to sensitive pediatric patient information.
